# Promotion of Physical Activity Using Point-of-Decision Prompts in Berlin Underground Stations

**DOI:** 10.3390/ijerph7083063

**Published:** 2010-08-04

**Authors:** Falk Müller-Riemenschneider, Marc Nocon, Thomas Reinhold, Stefan N. Willich

**Affiliations:** Institute for Social Medicine, Epidemiology and Health Economics, Charite Charite University Medical Centre Berlin, Luisenstrasse 57, 10098 Berlin, Germany; E-Mails: marc.nocon@charite.de (M.N); thomas.reinhold@charite.de (T.R); stefan.willich@charite.de (S.N.W)

**Keywords:** prevention, health promotion, physical activity, point-of-decision prompts

## Abstract

To evaluate point-of-decision prompts in the promotion of stair use in Germany, motivational posters were placed at three underground stations in Berlin. The proportion of passengers using stairs or stairways was counted before, during installation, and two weeks after removal of posters. In total, 5,467 passersby were counted. Stair use increased significantly in women, but not in men. The present pilot study thereby shows that the use of point-of-decision prompts is also feasible in Germany and it provides some evidence of effectiveness. Methodologically rigorous studies are warranted to confirm these findings.

## 1. Introduction

Physical inactivity is a significant risk factor for numerous chronic diseases such as diabetes, hypertension, cardiovascular and cerebrovascular diseases, malignancies, osteoporosis, injuries caused by falls and premature mortality [1–6]. Current guidelines therefore recommend being physically active on a regular basis. The prerequisite for a positive health effect is burning an additional 1,000 kcal per week through physical activity, or rather engaging in a minimum of 30 minutes of moderate physical activity on five days, or 20 minutes of vigorous physical activity three days per week [7,8]. Despite this knowledge, physical inactivity and sedentary lifestyle are major problems in industrialised nations [9–11].

In addition to the health consequences of physical inactivity and sedentary lifestyle, associated diseases reflect a considerable economic burden for health care systems [12]. Increasing physical activity through leisure-time activities, structured exercise or sports is therefore of importance to prevent the occurrence of many diseases and associated health care expenditures. Our environments, however, make performing exercises in our day to day lives difficult. Often the surroundings are arranged in such a way as to explicitly discourage engaging in physical activity, e.g., use of escalators and lifts instead of the stairs in train stations. Research in some industrialised nations has shown that it is possible to increase the usage of stairs in the aforementioned settings by using simple and fairly inexpensive posters and signs, so-called “point-of-decision prompts” [13]. Although this strategy may not increase physical activity sufficiently for any individual person to meet current recommendations, it could still have important public health implications. For instance, recent studies have shown, that on a population level, relatively small energy gaps from approximately 8 to 100 kcal per day are responsible for weight gains over past years [14–17]. By small increases in energy expenditure through stair use, point-of-decision prompts could therefore contribute to the improvement of energy balance on a population level.

Studies from different industrialized countries have investigated the effectiveness of point-of-decision prompts in different settings [13]. However, no investigations have yet been conducted in Germany. Also, it has been suggested, that intervention effectiveness might not be uniform across different target groups, settings, and types of prompts [13,18–22]. The objective of this pilot study was therefore, to test the feasibility and effectiveness of using posters as point-of-decision prompts in Germany. Specifically, this was done by investigating whether there was an increase in the usage of stairs among men and women in underground stations in Berlin as a result of exposure to point-of-decision prompts.

## 2. Methods

**Figure 1.**
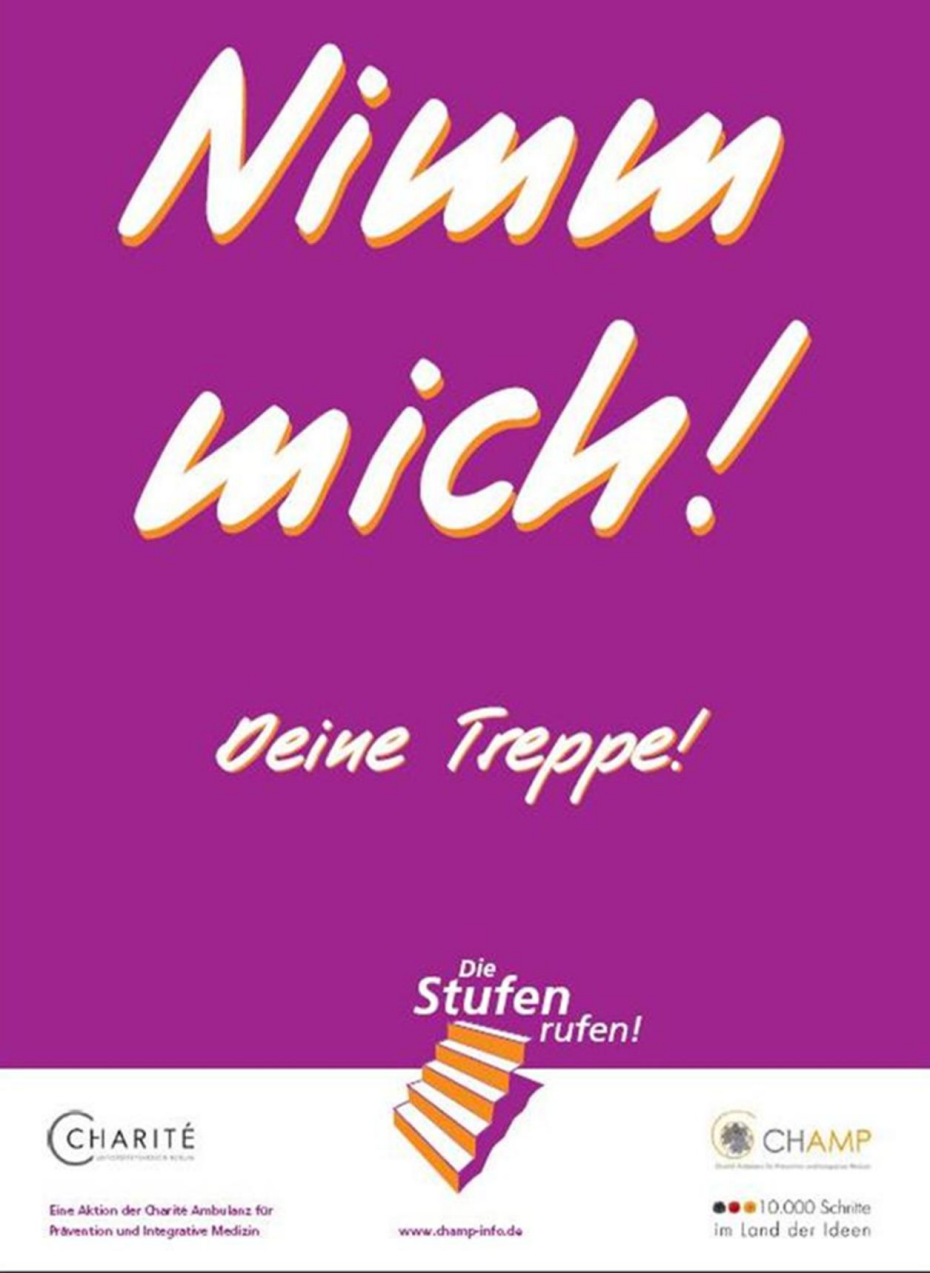
Poster design (translation of poster title “Take Me! Your Stairs!”).

The investigation was carried out as a pre- and post intervention study design. A poster was developed as a point-of-decision prompt. The format of the posters was A1 (594 × 841 mm) and they contained a message encouraging passersby to use the stairs instead of the escalators. The poster was developed by a team of marketing experts and researchers in the field of health promotion and prevention. The design of the posters is depicted in Figure 1. Three busy Berlin underground stations, that have both an escalator and a flight of stairs directly next to each other, were chosen. One poster was fastened at the bottom of each stairwell to facilitate ascent. Positions next to stairs and escalators were chosen in a way, to make them easily visible for passersby approaching stairs and escalators. The number of passersby that used either the stairs or the escalator for ascent where counted at four points in time.

Men and women were counted separately to investigate whether there were any sex specific differences. To minimize seasonal influences the study was conducted during 10 weeks in the period between August and October 2008. The first count was done before the posters were set up. The second count was done in the first week after the posters were up and the third count was done in the fifth week after the posters were up. The last count was done after 10 weeks, two weeks after the posters had been removed. All counts lasted one hour per underground station and were conducted during the morning rush hour. The counts were conducted on the same weekday, at the same time for each subway station.

The statistical analysis was done separately for men and women. We present the absolute numbers and numbers in percentages for stair and escalator use, for the four counts that were done. Further, the relative risk for the pre- and post-comparison are presented as well as the absolute and relative changes together with 95% confidence intervals. Two-sided tests of equal proportions were used to investigate differences in the proportion of stair users. P-values < 0.05 were considered statistically significant. Statistical analysis was performed using SAS version 9.2 (SAS Institute, Cary, NC, USA).

## 3. Results

In total 5,467 persons were counted, 3,167 women (58%) and 2,300 men (42%). The total number of men and women counted at different time points is presented in Tables 1 and 2. Before the posters were hung, 18.9% of the women (N = 164) and 29.6% of the men (N = 204) used the stairs. The remaining persons used the escalator. After the posters were up the number of women who used the stairs increased to 31.9% and decreased slightly after that, but stayed higher than before posters were hung, even in the 10^th^ week, two weeks after the posters were removed (Table 1). The difference compared to the baseline count is statistically significant for all further counts. There was no influence on the number of men who used the stairs at any point in time (Table 2). When considering men and women together, the proportion of persons who used the stairs was 23.7% at baseline and increased to 30.4%, 27.2%, and 30.0%, at week 1, week 5 and week 10, respectively.

## 4. Discussion

The population strategy to increase physical activity that was investigated in this pilot study was simple and cheap to implement. In summary, a significant increase in the use of stairs in women was noted and use remained at a higher level, even after the point-of-decision prompts had been removed. The change in use of stairs in women was, compared to the effect found in previous studies, considerable. Only a few previous studies have reported absolute increases in stair use as large as those found in women [13,21,22]. Remarkably, the use of stairs in women remained elevated, even after the prompts had been removed. This gives reason to believe that this type of population study can have an effect lasting longer than the intervention itself. Findings supporting this are found in previous studies that investigate the effectiveness of point-of-decision prompts. In a current systematic review Nocon *et al.* identified 10 studies that investigated the use of stairs after point-of-decision prompts had been removed. Of these 10 studies, seven showed that there was an effect after the removal of the posters [23]. However, point of decision prompts in our study, had no influence on the use of stairs in men. The results therefore show a sex specific effect that continued to exist after the intervention was over. Sex specific effects similar to these were also shown in previous studies, but with a nonuniform tendency [19,23–26].

Possible explanations for the lack of an effect in men include, that the message on the posters was not interesting to male passersby and therefore could not increase their motivation to use the stairs. It is also possible, however, that men are generally less receptive to appeals to change their behaviour and they would not have changed their behaviour, irrespective of the type of message that was posted. The sex specific difference could also be explained by the fact that men used the stairs more than women at the beginning of the study. An increase beyond the baseline use would therefore be harder to achieve in men. Similar results were also found in a previous study by Coleman *et al.* [21]. Other components that could influence effectiveness are, e.g., the visibility of the posters. This, however, is unlikely to be causal for a sex specific effect of point-of-decision prompts in our study, because positioning was chosen to allow for best possible visibility for passersby approaching escalators and stairs. In addition to the use of posters as point-of-decision prompts, some studies have investigated the effectiveness of other types of prompts, such as stairwell advertising, banners, posters of different sizes, different messages *etc.* They provided some evidence, that certain point-of-decision prompts could be more effective to increase stair use than others [18,27,28]. While this knowledge will help to tailor future interventions, further research will be necessary to investigate these questions [29].

When interpreting findings of this pilot study, certain limitations should be noted. The pre- and post-intervention study design, and the consequent lack of an adequate control group, is of special importance. The relevance of external factors and their influence on the measured effect can therefore not be accessed adequately. Therefore, it cannot be excluded that the low number of women who used the stairs at baseline was a chance finding. Considering this, the increase in stair use in women could be interpreted as an artefact. Although the consistency in stair use in men throughout the study contradicts this interpretation somewhat, a study with a suitable control group is essential to interpret the results correctly. However, previous studies from other countries, investigating the effectiveness of point-of-decision prompts were also pre- and post studies in the majority of cases, thereby suffering from similar limitations. Another limitation of the present study is the relatively small extent of the information that was collected. Information regarding the age and constitution of the passersby would have been of interest, as would the qualitative aspects of the perception and evaluation of the posters. Moreover, it could be of great interest to investigate the effectiveness of this population strategy independently of regional characteristics. Selecting suitable underground stations in socioeconomic diverse regions, in different cities, and in different federal states would therefore be of great interest. In addition, the use of point-of-decision prompts could also be investigated in other environments, such as shopping malls and office buildings, where baseline rates of stair climbing might be different. The effectiveness of point-of-decision prompts in these other settings might therefore also differ from that in underground stations.

In summary, the present pilot study is, to our knowledge, the first that investigates point-of-decision prompts in Germany. It shows that use of point-of-decision prompts is feasible and provides some evidence that population strategies of this type can be associated with positive and meaningful behaviour changes. To confirm these findings and to identify the optimal type of message, further and methodologically rigorous investigations are warranted. These should include a control group study design, preferably a randomised controlled trial, including an economic evaluation in order to investigate effectiveness and cost-effectiveness of this preventive population strategy.

## Figures and Tables

**Table 1.** Stair use of women at different points in time.

**N=3,167**	**Without poster Baseline (n = 867)**	**With poster Week 1 (n = 796)**	**With poster Week 5 (n = 656)**	**Without poster Week 10 (n = 848)**
Use of stairs	18.9% 164	31.9% 254	28.5% 187	31.5% 267
Absolute change* (95% CI)	-	13.0%** (8.8–17.1)	9.6%** (5.3–13.9)	12.6%** (8.5–16.6)
Relative change* (95% CI)	-	68.7% (46.6–90.6)	50.7% (27.9–73.7)	66.5 (44.8–87.8)
Relative Risk* (95% CI)	-	1.69 (1.42–2.00)	1.51 (1.25–1.81)	1.66 (1.40–1.97)

* compared to baseline count,

** p < 0.0001, CI: confidence interval.

**Table 2.** Stair use of men at different points in time.

**N = 2,300**	**Without poster Baseline (n = 690)**	**With poster Week 1 (n = 577)**	**With poster Week 5 (n = 449)**	**Without poster Week 10 (n = 584)**
Use of stairs	29.6% 204	28.4% 164	25.4% 114	27.7% 162
Absolute change* (95% CI)	-	−1.1%** (−6.1–3.9)	−4.2%** (−9.4–1.2)	−1.8%** (−6.8–3.2)
Relative change * (95% CI)	-	−3.9% (−20.1–13.2)	−14.1% (−31.6–4.0)	−6.2% (−22.9–10.7)
Relative Risk* (95% CI)	-	0.96 (0.81–1.14)	0.86 (0.71–1.04)	0.94 (0.79–1.12)

* compared to baseline count,

** p > 0.05, CI: confidence interval.

## References

[1] Warburton DE Nicol CW Bredin SS Health benefits of physical activity: the evidence CMAJ 2006 174 801 809 16534088 10.1503/cmaj.051351 PMC1402378

[2] Nocon M Hiemann T Müller-Riemenschneider F Thalau F Roll S Willich SN Association of physical activity with all-cause and cardiovascular mortality: a systematic review and meta-analysis Eur. J. Cardiovasc. Prev. Rehabil 2008 15 239 246 18525377 10.1097/HJR.0b013e3282f55e09

[3] Hu FB Stampfer MJ Colditz GA Ascherio A Rexrode KM Willett WC Manson JE Physical activity and risk of stroke in women JAMA 2000 283 2961 2967 10865274 10.1001/jama.283.22.2961

[4] Hu FB Manson JE Stampfer MJ Colditz G Liu S Solomon CG Willett WC Diet, lifestyle, and the risk of type 2 diabetes mellitus in women N. Engl. J. Med 2001 345 790 797 11556298 10.1056/NEJMoa010492

[5] Camacho TC Roberts RE Lazarus NB Kaplan GA Cohen RD Physical activity and depression: evidence from the Alameda County Study Am. J. Epidemiol 1991 134 220 231 1862805 10.1093/oxfordjournals.aje.a116074

[6] Lee IM Hsieh CC Paffenbarger RS Jr Exercise intensity and longevity in men. The Harvard alumni health study JAMA 1995 273 1179 1184 7707624

[7] Warburton DE Nicol CW Bredin SS Prescribing exercise as preventive therapy CMAJ 2006 174 961 974 16567757 10.1503/cmaj.1040750 PMC1405860

[8] Haskell WL Lee IM Pate RR Powell KE Blair SN Franklin BA Macera CA Heath GW Thompson PD Bauman A Physical activity and public health: updated recommendation for adults from the American College of Sports Medicine and the American Heart Association Circulation 2007 116 1081 1093 17671237 10.1161/CIRCULATIONAHA.107.185649

[9] Brownson RC Boehmer TK Luke DA Declining rates of physical activity in the United States: what are the contributors? Annu. Rev. Public Health 2005 26 421 443 15760296 10.1146/annurev.publhealth.26.021304.144437

[10] Lampert T Mensink GB Ziese T Sport and health among adults in Germany [in German] Bundesgesundheitsblatt Gesundheitsforschung Gesundheitsschutz 2005 48 1357 1364 16270185 10.1007/s00103-005-1169-4

[11] Owen N Bauman A The descriptive epidemiology of a sedentary lifestyle in adult Australians Int. J. Epidemiol 1992 21 305 310 1428485 10.1093/ije/21.2.305

[12] Muller-Riemenschneider F Reinhold T Berghofer A Willich SN Health-economic burden of obesity in Europe Eur. J. Epidemiol 2008 23 499 509 18509729 10.1007/s10654-008-9239-1

[13] Kahn EB Ramsey LT Brownson RC Heath GW Howze EH Powell KE Stone EJ Rajab MW Corso P The effectiveness of interventions to increase physical activity. A systematic review Am. J. Prev. Med 2002 22 73 107 11985936 10.1016/s0749-3797(02)00434-8

[14] Hill JO Wyatt HR Reed GW Peters JC Obesity and the environment: where do we go from here? Science 2003 299 853 855 12574618 10.1126/science.1079857

[15] Zhai F Wang H Wang Z Popkin BM Chen C Closing the energy gap to prevent weight gain in China Obes Rev 2008 9 Suppl. 1 107 112 18307711 10.1111/j.1467-789X.2007.00450.x

[16] Brown WJ Williams L Ford JH Ball K Dobson AJ Identifying the energy gap: magnitude and determinants of 5-year weight gain in midage women Obes. Res 2005 13 1431 1441 16129726 10.1038/oby.2005.173

[17] Berg C Rosengren A Aires N Lappas G Toren K Thelle D Lissner L Trends in overweight and obesity from 1985 to 2002 in Goteborg, West Sweden Int. J. Obes. (Lond.) 2005 29 916 924 15852045 10.1038/sj.ijo.0802964

[18] Olander EK Eves FF Puig-Ribera A Promoting stair climbing: stair-riser banners are better than posters... sometimes Prev. Med 2008 46 308 310 18155757 10.1016/j.ypmed.2007.11.009

[19] Webb OJ Eves FF Effects of environmental changes in a stair climbing intervention: generalization to stair descent Am. J. Health Promot 2007 22 38 44 17894262 10.4278/0890-1171-22.1.38

[20] Webb OJ Eves FF Promoting stair climbing: intervention effects generalize to a subsequent stair ascent Am. J. Health Promot 2007 22 114 119 18019888 10.4278/0890-1171-22.2.114

[21] Coleman KJ Gonzalez EC Promoting stair use in a US-Mexico border community Am. J. Public Health 2001 91 2007 2009 11726384 10.2105/ajph.91.12.2007 PMC1446923

[22] Russell WD Dzewaltowski DA Ryan GJ The effectiveness of a point-of-decision prompt in deterring sedentary behavior Am. J. Health Promot 1999 13 257 259 10538638 10.4278/0890-1171-13.5.257

[23] Nocon M Müller-Riemenschneider F Nitzschke K Willich SN Increasing physical activity with point-of-choice prompts—A systematic review Scand J Public Health 2010 (epub ahead of print) 10.1177/1403494810375865 20601438

[24] Kerr J Eves F Carroll D Can Posters Prompt Stair Use in a Worksite Environment? J. Occup. Health 2001 43 205 207

[25] Eves FF Webb OJ Mutrie N A workplace intervention to promote stair climbing: greater effects in the overweight Obesity 2006 14 2210 2216 17189548 10.1038/oby.2006.259

[26] Boutelle KN Jeffery RW Murray DM Schmitz MK Using signs, artwork, and music to promote stair use in a public building Am. J. Public Health 2001 91 2004 2006 11726383 10.2105/ajph.91.12.2004 PMC1446922

[27] Kerr J Eves FF Carroll D The influence of poster prompts on stair use: The effects of setting, poster size and content Br. J. Health Psychol 2001 6 397 405 12614513 10.1348/135910701169296

[28] Kerr J Eves F Carroll D Encouraging Stair Use: Stair-Riser Banners Are Better Than Posters Am. J. Health Promot 2001 91 1192 1193 10.2105/ajph.91.8.1192 PMC1446744 11499102

[29] Webb OJ Eves FF Promoting stair use: Single *versus* multiple stair-riser messages Am. J. Public Health 2005 95 1543 1544 16051937 10.2105/AJPH.2004.046235 PMC1449395

